# Epidemical and etiological study on hand, foot and mouth disease following EV-A71 vaccination in Xiangyang, China

**DOI:** 10.1038/s41598-020-77768-7

**Published:** 2020-12-01

**Authors:** Xiao-Dan Meng, Yeqing Tong, Zhen-Ni Wei, Lei Wang, Jian-Yi Mai, Yang Wu, Zhi-Yu Luo, Shaoping Li, Meng Li, Siquan Wang, Sheng Wei, Wensheng Gong, Wangsheng Zhang, Xingzhou Hu, Jiao Huang, Jing Shi, Gang Yang, Shengli Meng, Zejun Wang, Xuhua Guan, Shuo Shen

**Affiliations:** 1 Wuhan Institute of Biological Products Co. Ltd, Wuhan, 430207 China; 2 Hubei Provincial Center for Diseases Control and Prevention, North Road 6# of Hongshan District, Wuhan, 430079 Hubei China; 3 Xiangyang Center for Disease Control and Prevention, Xiangyang, 441021 China; 4 School of Public Health, Tongji Medical College, Huazhong University of Science and Technology, Wuhan, 430030 China; 5 National Engineering Technology Research Center of Combined Vaccines, No.1 Huangjin Industrial Park, Wuhan, 430207 Hubei China

**Keywords:** Microbiology, Molecular biology, Medical research

## Abstract

Coxsackievirus A6 (CV-A6) and Coxsackievirus A10 (CV-A10) have been emerging as the prevailing serotypes and overtaking Enterovirus A71 (EV-A71) and Coxsackievirus A16 (CV-A16) in most areas as main pathogens of hand, foot and mouth disease (HFMD) in China since 2013. To investigate whole etiological spectrum following EV-A71 vaccination of approximate 40,000 infants and young children in Xiangyang, enteroviruses were serotyped in 4415 HFMD cases from October 2016 to December 2017 using Real Time and conventional PCR and cell cultures. Of the typeable 3201 specimen, CV-A6 was the predominant serotype followed by CV-A16, CV-A10, CV-A5, CV-A2 and EV-A71 with proportions of 59.54%, 15.31%, 11.56%, 4.56%, 3.78% and 3.03%, respectively. Other 12 minor serotypes were also detected. The results demonstrated that six major serotypes of enteroviruses were co-circulating, including newly emerged CV-A2 and CV-A5. A dramatic decrease of EV-A71 cases was observed, whereas the total cases remained high. Multivalent vaccines against major serotypes are urgently needed for control of HFMD.

## Introduction

Hand, foot and mouth disease (HFMD) is a common children’s illness in countries of Asia-Pacific region. Currently it was mainly caused by infection of four serotypes EV-A71 (Enterovirus A71), CV-A16 (Coxsackievirus A16), CV-A6 (Coxsackievirus A6) and CV-A10 (Coxsackievirus A10) of human enterovirus (HEV) A species in China, although members of B, C and even D species were also detected less frequently or occasionally in specimens of HFMD patients^1,2^. The disease is characterized by fever, sore throat, general malaise, and vesicular eruptions on the hands, feet, oral mucosa, and tongue. Severe cases involve complications such as encephalitis, meningitis, acute flaccid paralysis, cardiorespiratory failure, brainstem encephalitis leading to death. EV-A71 has been responsible for the most severe and fatal cases reported, while other serotypes also cause severe cases in less frequencies^3^. For example, between 2008 and 2012, a large scale of national surveillance in China revealed that HFMD were mainly caused by EV-A71 (40%) and CV-A16 (40%)^4^. EV-A71 caused more than 90% of severe and fatal cases, while non-EV-A71 enteroviruses were associated with less than 10% of severe and fatal cases. In another large scale of investigation including 21,615 confirmed HEV infections, among 389 severe cases, 249 were caused by EV-A71 (64.1%), 13 by CV-A16 (3.3%) and 127 by non-EV-A71/non-CV-A16 (32.6%), respectively^5^.

HEVs are classified into four type species (A, B, C and D) in the genus *enterovirus* within the family *Picornaviridae.* Each species includes multiple serotypes and each serotype was further divided into genotypes, subgenotypes and lineages (or clades and clusters)^6^. Currently 106 different HEV serotypes have been classified, historically based on cross-neutralization of antibodies, sensitive to grow in different cells and to infect suckling mouse, now largely based on homology of the VP1 sequence^7^. Enterovirus contains a single stranded, positive sense RNA genome approximately 7.4 kb in length. HEVs have been involving rapidly and recombination occurred frequently. Wide spectrum of diseases and genetic diversity make it difficult for vaccine development. It has been reported that mutation and recombination may be the reasons of emerging and dominance of CV-A6 variants^8^ and are increasing the likelihood of severe infection cases^9^.

After 2012 in China or even earlier in some areas in China and other countries, CV-A6 and CV-A10 have become the predominant enterovirus serotypes, overtaking EV-A71 and CV-A16, causing mild, severe and fatal cases of HFMD^10–12^. EV-A71 vaccine has been available since 2016 and is expected to reduce mild, severe and fatal cases dramatically. However, for complete control of HFMD caused by many serotypes, development of multivalent vaccines is urgently needed. Epidemic and etiological investigations provide information on control measures and formulations of multivalent vaccines against HFMD, especially following the vaccination of monovalent EV-A71 vaccine. Characterization and molecular biology studies on cell-isolated enteroviruses are essential for development of inactivated vaccine and therapeutics.

In this study, three approaches, fluorescence quantitative real-time polymerase chain reaction (Real Time RT-PCR), conventional RT-PCR amplification/sequencing of genome fragments and cell cultures were combined and were used for serotyping/genotyping and characterization of HEVs in HFMD specimens. The aims of the study were to enroll as many as HFMD patients in 15 months in Xiangyang, China, to serotype the enteroviruses in specimen and to investigate full etiological spectrum following a large scale of vaccination of EV-A71. The results showed that as many as 18 enterovirus serotypes were detected and 188 strains were isolated from all detected serotypes in RD and/or Vero cells. CV-A6 was predominating, CV-A10 and CV-A16 were continuously prevailing and EV-A71 proportion was dramatically decreased. Other 14 serotypes were also detected. Interestingly, CV-A2 and CV-A5 were emerging as two main serotypes, co-circulating together with other four well-recognized pathogens. The multiple serotype feature of pathogens becomes a challenging issue for HFMD control. The results demonstrate that surveillance, serotyping and genetic characterization of co-circulating HEVs are conducive to understand the epidemic of HFMD and to monitor the changes of etiological spectrum, as well to contribute to development of multivalent vaccines and strategies for HFMD control.

## Results

### Detection of HEVs and serotyping of EV-A71 specimens from patients of HFMD by real time RT-PCR

**Figure 1:**
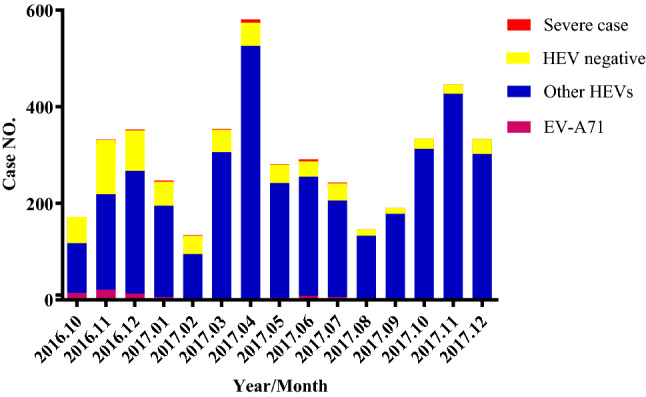
Monthly distribution of total and severe HFMD cases Total number of HFMD cases caused by EV-A71, other HEVs and non-HEV (HEV negative) were 4415 and were confirmed using Real Time PCR Kits, specific EV-A71 and pan-HEVs.

Total RNAs were extracted from rectal swabs obtained from patients of HFMD and were used for detection of EV-A71 and other HEVs by Real Time RT-PCR. As shown in Fig. 1, among 4415 cases of HFMD, 3781 (85.64%) were caused by enteroviruses, including 78 cases caused by EV-A71 (1.77%) and 3703 (83.87%) by non-EV-A71 enteroviruses (EV-A71−/HEV+). Fatal cases were not observed and 22 severe cases occurred. The rest of 634 cases (14.36%) were negative for enterovirus RNAs determined by using Real Time RT-PCR. It seems that other viral pathogens might be associated with the symptoms of HFMD, which should be further investigated. In human intestine there are many viruses which may not related to HFMD and detected by chances^13^. Alternatively, the pan-enterovirus Real Time RT-PCR kits used may not be sensitive to all HEVs equally.

### Demographic data

Monthly distribution (Fig. 1) showed that there were two epidemic peaks a year, November to December and March each year. Twenty-two severe cases occurred even after the large scale vaccination of EV-A71 vaccine in September and October in 2016. These cases were caused by non-EV-A71 enteroviruses. Gender distribution revealed that more male than female children were diagnosed as HFMD, accounting for 59.05% (2607/4415) for the male and 40.95% (1808/4415) for the female patients, respectively. Interestingly, it was observed that after two doses of EV-A71 vaccination of nearly 40,000 infants and children (32.59% of vaccination of targeted population in Xiangyang) in September and October in 2016, The HFMD cases caused by EV-A71 were dramatically decreased from the beginning to the end of 2017, The details information can be found as Supplementary Table S1 online.

Age distribution indicated that the most cases occurred in the age groups of 1– < 2, 2– < 3 and 3– < 4 years old with percentages of 38.62% (1705/4415), 23.06% (1018/4415) and 20.75% (916/4415), respectively. A few cases were observed in the groups of 0– < 1 and 4– < 5 years old, while children 5 or more than 5 years old were less frequently infected with HFMD associated HEVs.

### Serotyping of EV-A71−/HEV+ specimens by conventional RT-PCR

**Figure 2:**
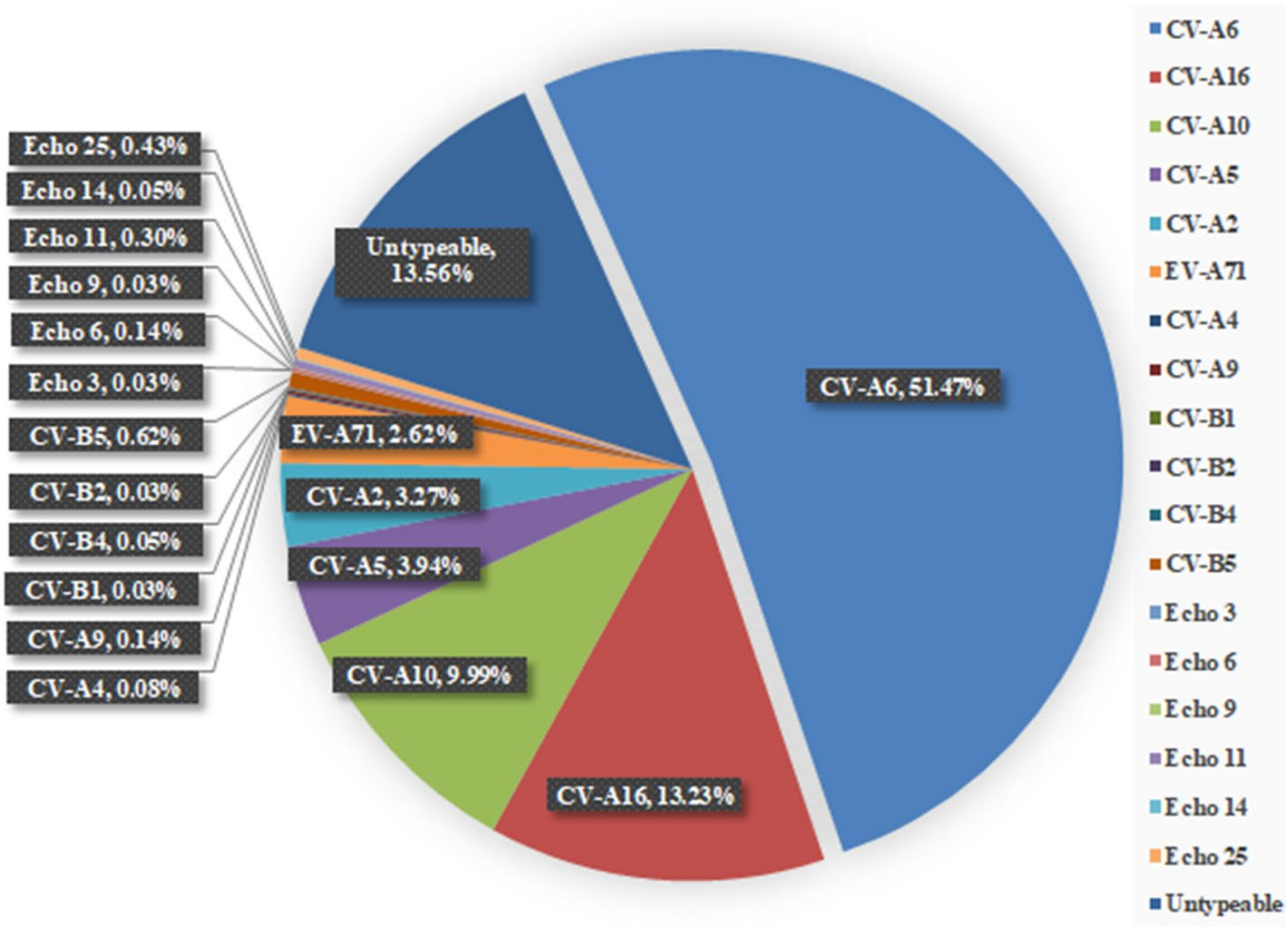
Proportion of the untypeable and typeable enteroviruses identified in HFMD patients. The total numbers of 3703 specimens were used for serotyping with combination of real time RT-PCR, conventional RT-PCR and cell cultures.

For further serotyping/genotyping of EV-A71−/HEV+ specimens and CPE positive specimens of cell isolates, the sequences of the 5′-UTR were amplified by conventional RT-PCR first using pan-HEV primers. The genome fragments of untypeable specimens were then further amplified using the VP1-specific pan-HEV primers to increase ratio of the typeable HEVs. As shown in Fig. 2, of 3703 specimens, 86.44% of HEVs (3201/3703) were genotyped by blasting and comparison with sequences of enteroviruses in GenBank. The typeable case number was 3201. The percentage of untypeable HEV samples was 13.56% (502/3703).

It was noted that 19 more EV-A71 were identified which were not detected using EV-A71 specific Real Time RT-PCR Kit mentioned above. For EV-A71, 78 cases were confirmed by Real Time RT-PCR, and 19 cases were confirmed by conventional RT-PCR and sequencing of the 5′-UTR and the VP1 gene fragments.

**Figure 3:**
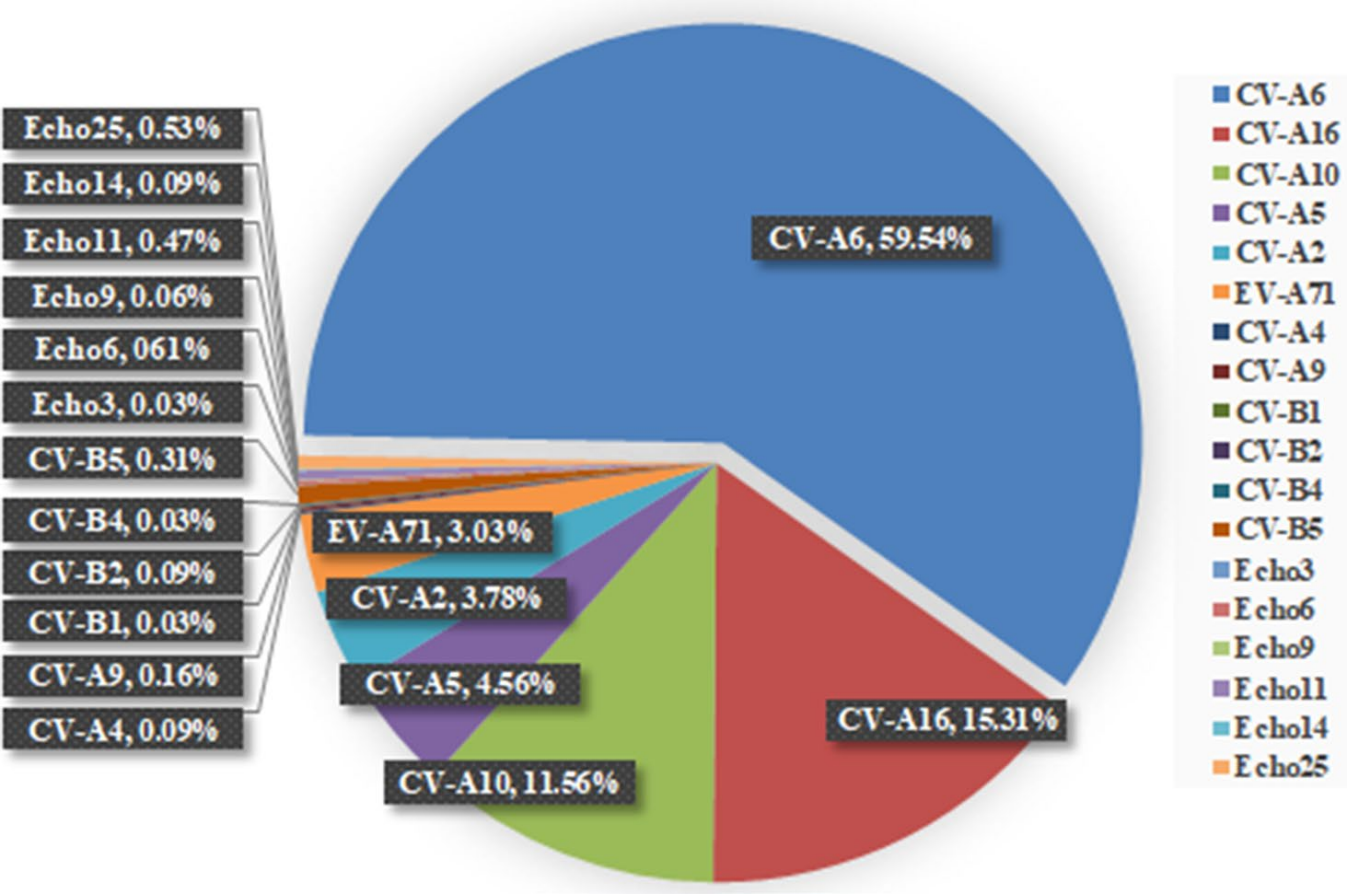
Proportion of the confirmed enterovirus serotypes identified in HFMD patients. The total case numbers of confirmed serotypes were 3201 performed with combination of Real Time RT-PCR, conventional RT-PCR and cell cultures.

As shown in Fig. 3 and summarized in Table 1, among the serotyped 3201 cases of HFMD, CV-A6 was the major and predominating serotype followed by CV-A16, CV-A10, CV-A5, CV-A2 and EV-A71 and the ratios were 59.54% (1906/3201), 15.31% (490/3201), 11.56% (370/3201), 4.56% (146/3201), 3.78% (121/3201) and 3.03% (97/3201), respectively. They were main serotypes and co-circulating. Twelve minor serotypes CV-A4 (3/3201), CV-A9 (5/3201), CV-B1 (1/3201), CV-B2 (1/3201), CV-B4 (2/3201), CV-B5 (23/3201), Echoviruses 3 (1/3201), Echoviruses 6 (5/3201), Echoviruses 9 (1/3201), Echoviruses 11 (11/3201), Echoviruses 14 (2/3201) and Echoviruses 25 (16/3201) were also identified but they accounted only for 2.22% (71/3201) together.

**Table 1:** Serotyping, cell isolation of enteroviruses associated with HFMD.

Serotypes	No. of samples	%	No. and % of cell isolation					Species
			RD+	%*	Vero+	%*	No. of strains	
CV-A6	1906	59.54	9	0.47	0	0.00	9	A
CV-A16	490	15.31	3	0.61	68	13.88	71	A
CV-A10	370	11.56	11	2.97	0	0.00	11	A
CV-A5	146	4.56	3	2.05	0	0.00	3	A
CV-A2	121	3.78	9	7.44	0	0.00	9	A
EV-A71#	97	3.03	1	1.03	7	7.22	8	A
CV-A4	3	0.09	1	33.33	0	0.00	1	A
CV-A9	5	0.16	1	20.00	1	20.00	2	B
CV-B1	1	0.03	0	0.00	1	100.00	1	B
CV-B2	1	0.03	0	0.00	1	100.00	1	B
CV-B4	2	0.06	2	100.00	4	200.00	6	B
CV-B5	23	0.72	14	60.87	9	39.13	23	B
Echo3	1	0.03	1	100.00	0	0.00	1	B
Echo6	5	0.16	5	100.00	0	0.00	5	B
Echo9	1	0.03	1	100.00	0	0.00	1	B
Echo11	11	0.34	15	136.36	0	0.00	15	B
Echo14	2	0.06	3	150.00	0	0.00	3	B
Echo25	16	0.50	18	112.50	0	0.00	18	B
No. of strains	3201		97		91		188	A and B

*More than 100% of isolation rates were caused by co-infection of patients with two enteroviruses. ^#^ Nineteen EV-A71 cases were detected by conventional RT-PCR and 78 were detected by Real Time RT-PCR.

**Figure 4:**
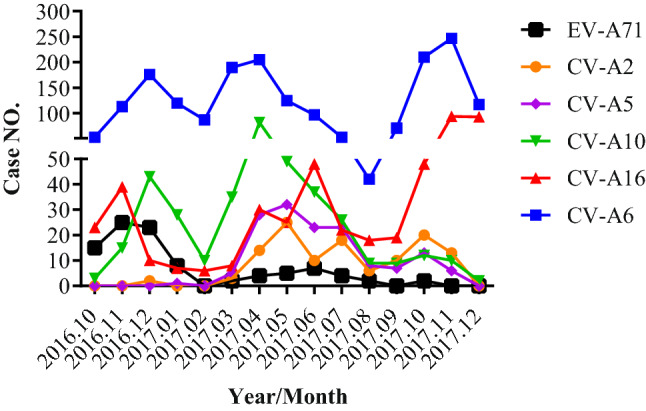
Monthly distribution of HFMD cases caused by the six major serotypes. A total number of HFMD cases caused by the six major serotypes is 3130, confirmed with combination of Real Time RT-PCR, conventional RT-PCR and cell cultures.

Monthly distribution of the six main serotypes was analyzed and shown in Fig. 4. It was found that they are co-circulating in 7 out of 12 months in 2017, from March to August and in October. The proportions of CV-A2 and CV-A5 in these 7 months reached to 5.91% and 8.12%, respectively. In October CV-A2 was the third largest pathogen, and in May and July CV-A5 became the third largest pathogen in term of case numbers, the details information can be found as Supplementary Table S2 online.

There were 22 severe cases. Among them, 12 were typeable and 8 were untypeable HEVs (Table 2), and rectal swabs from two patients were not obtained. Severe cases were caused by CV-A6, CV-A10, CV-A2, CV-A5 and EV-A71 at ratios of 0.31%, 0.54%, 1.65%, 0.68% and 1.03%, respectively. It demonstrated that non-EV-A71 also caused severe cases and CV-A2 was at even higher ratio than that of EV-A71. The EV-A71-patient was not vaccinated, while other patients were either vaccinated or not vaccinated with inactivated EV-A71 vaccine, indicating EV-A71 vaccination did not protect severe disease caused with non-EV-A71. Gender distributions showed that 13 were male and 7 were female, while 13, 6 and 1 severe cases occurred in groups of 0– < 1, 1– < 2 and ≥ 2 years old, respectively.

**Table 2:** Serotypes causing severe cases with or without EV-A71 vaccination.

Serotypes	CV-A6	CV-A10	CV-A2	CV-A5	EV-A71	Untypeable
Severe case No	6	2	2	1	1	8
Total case No	1906	370	121	146	97	502
Ratio (%)	0.31	0.54	1.65	0.68	1.03	1.59
Vaccination/not	2/4	0/2	1/1	0/1	0/1	2/6

### Isolation of HFMD associated enteroviruses in RD and vero cells

To further characterize and serotype these HFMD-associated HEVs, 3494 EV-A71-/HEV+ specimens were inoculated in monolayer of RD and Vero cells for virus isolation and cell tropism study. The viruses were blindly passaged for 3 times, the CPE positive cultures were passaged once more and serotypes were determined by sequence analysis of both the 5′-UTR and the VP1 regions. As shown in Table 1, 188 CPE positive isolates were obtained and genotyped. It was interesting that six serotypes were isolated which were not detected by Real Time RT-PCR and conventional PCR.

It was noticed that from EV-A71−/HEV+ specimens originally determined by Real Time RT-PCR, 19 EV-A71 were detected by conventional RT-PCR/sequencing as mentioned above, and among them 9 EV-A71 strains were also isolated in cells. It was not clear if cell cultures were more sensitive than Real Time RT-PCR for identifying these EV-A71 strains. It was also interesting that 28 enteroviruses were isolated in cells from EV-A71−/HEV+ specimens, which were untypeable as genome fragments of these HEVs were not amplified by conventional RT-PCR. This indicated that it was still possible to isolate the viruses in cell cultures even if the viral genome fragments were not amplified by RT-PCR.

It was found that much more kinds of serotypes were isolated in RD cells than in Vero cells (at a ratio of 17:7). EV-A71 and CV-A16 were much easily isolated in Vero cells than in RD cells (Vero/RD isolating ratios, 7/1 and 68/3, respectively) with high isolation ratios of 36.9% (7/19) and 13.9% (68/490), respectively (only a portion of EV-A71 specimens were used for cell isolation). The predominant CV-A6 and CV-A10 were difficult to be isolated in these two cells, especially in Vero cells. The ratios of cell isolations were 0.47% (9/1907) for CV-A6 and 2.97% (11/370) for CV-A10, all of them were isolated only in RD cells.

The total percentage of isolation was 5.38% (188/3494). The isolation rate was 2.78% (97/3494) in RD cells and 2.63% in Vero cells (91/3494). Some minor serotypes were much more easily isolated in RD cells. For example, one CV-A4 and six different echoviruses were isolated in RD cells, but none of them were isolated in Vero cells. On the other hand, CV-B1, -B2, -B4 and -B5 were more easily isolated in Vero cells or RD cells. CV-A9 was isolated in both Vero and RD cells.

### Co-infection by two serotypes

If a cell isolate was a different serotype compared to that of its original rectal swab, it was considered as a co-infection by two enteroviruses. To keep the total number of cases unchangeable, serotype for each co-infection sample was counted as that of cell cultured strain. As shown in Table 3, predominating CV-A6 co-infected with one of other 10 different serotypes, accounting for 32 out of 45 co-infections. On the other hand, seven of each CV-B5, Echovirus 11 and 25 co-infected with CV-A6, demonstrating again that CV-B5, Echovirus 11 and 25 (also other species B HEV) were much more easily isolated in RD cells than the CV-A6. All EV-A71, CV-A16, CV-A6, and CV-A10 cell isolates were also confirmed by neutralization assays using in-house prepared rabbit antisera against these four enteroviruses.

**Table 3:** Numbers of co-infection of patients with two serotypes determined by gene sequencing of swabs and cell-isolates.

Cell isolate VP1 sequence	Viral sequence of swabs (5′-UTR and/or VP1)							No. of isolates
	CV-A6	CV-A10	CV-A16	EV-A71	CV-A5/CV-A16	Echo9/11	Echo14	
EV-A71	1							1
CV-A2	1							1
CV-A16	3					1		4
CV-B1	1							1
CV-B2	1							1
CV-B5	7	1					1	9
Echo6	2					1		3
Echo11	7	1	1		1	1		11
Echo14	2							2
Echo25	7	2	2	1				12
No. of co-infection	32	4	3	1	1	3	1	45

Ten isolates in RD or Vero cells were untypeable using pan-enterovirus primers for amplification of the 5′-UTR and the VP1 region (data not shown). The CPE in these cultures may be caused by other non-HEV viruses which accidentally co-existed in specimens of HEV positive HFMD patients. Another possibility is that pan-HEV primers or probes used were not sensitive enough for detection of some HEV serotypes.

### Phylogenetic analysis of the six main serotypes

**Figure 5:**
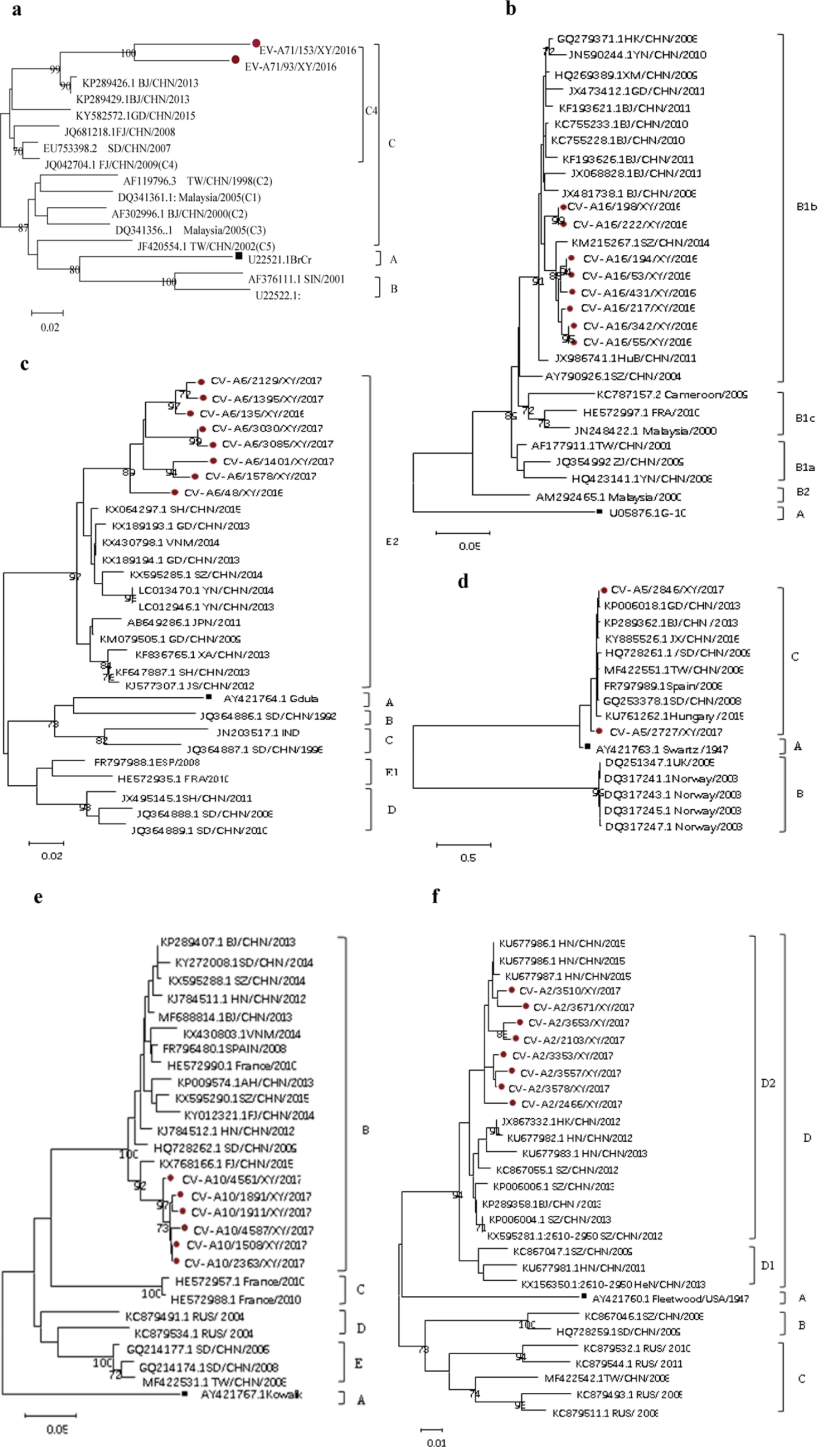
Phylogenetic dendrograms based on the partial VP1 nucleotide sequences of EV-A71 (**a**), CV-A16 (**b**), CV-A6 (**c**), CV-A5 (**d**), CV-A10 (**e**), CV-A2 (**f**). The dendrograms were constructed using the neighbor-joining method based on the alignment of the partial VP1-region sequences of Xiangyang strains and other strains downloaded from GenBank. Bootstrap values (%) for 1000 replicates were calculated, and only values > 70% are shown at the nodes. Red dots indicate Xiangyang strains, and black square indicate prototype strains.

Phylogenetic analysis of CV-A6, CV-A16, CV-A10, CV-A5, CV-A2 and EV-A71 were performed based on the partial VP1 sequences (Fig. 5). The sequences of each serotype were aligned with the reference sequences retrieved from the GenBank. The results show that CV-A6, CV-A16, CV-A10, CV-A5, CV-A2 and EV-A71 detected in Xiangyang belong to genotypes (or subgenotypes and clades) E2, B1b, B, C, D2 and C4, respectively^9,11,14–19^. They are clustered with dominate, circulating genotypes/subgenotypes identified recently in different areas in China, and have long genetic distances from each of the corresponding prototypes. Based on the sequences of the partial 5′-UTR and VP1, it was found that the same subgenotypes of each individual serotypes were circulating in Xiangyang. The analysis based on the 5′-UTR revealed the similar results (data not shown).

## Discussion

To date, 106 HEVs have been discovered^6^ and, at least, more than 36 enterovirus serotypes have been detected in clinical samples of HFMD or herpangina (HA) patients^9,20,21^ in China, though most of them are occasionally detected at much low proportions, indicating sporadic infection. Recently CV-A6, CV-A10, CV-A16 and EV-A71 are the predominating etiological agents and some of other serotypes, such as CV-A2, CV-A4 and CV-A5, were also reported to cause sporadic HFMD cases or epidemic of herpangina. Numerous studies showed that EV-A71 was responsible for majority of severe and fatal cases. It was a rare event that both CV-A2 and CV-A5 emerged at high ratios as two of the six main serotypes for HFMD epidemic in Xiangyang following the large scale EV-A71 vaccination, co-circulating with CV-A6, CV-A16, CV-A10 and EV-A71 in 15-months surveillance. These two serotypes also caused severe cases of HFMD as found in this study and previous reports^22,23^. The potential relationship between EV-A71 vaccination and the emerging of these two serotypes is worthy to be further investigated. Further surveillance is warranted to determine whether vaccination of EV-A71 impart selective pressure for these two serotypes and other serotypes to emerge at high proportions.

Previous studies on epidemics revealed that CV-A2 were mainly involved in aseptic meningitis and HA herpangina, while CV-A5 also caused sporadic cases of HFMD. In an aseptic meningitis outbreak in Taiwan in 2006, Echo 30, CV-A2, CV-A5 and other enteroviruses were detected in specimens of patients^24^. For CV-A2, Chen and colleagues reported that CV-A2 was the predominant serotype in the enterovirus outbreak in Taiwan in 2008^25^. Of 280 hospitalized patients, 83.6% (183/280) of CV-A2-infected patients presented with herpangina, while 78.3% (97/280) of patients with EV-A71 infection presented with HFMD. Only 4.4% (8/280) of HFMD was caused by CV-A2. In both epidemics occurred in Taiwan, serotyping of enteroviruses was only performed on a portion of all cases. Central nervous system complications were found in 18.6% (18/97) of EV-A71-infected children, but only in 1.1% (2/183) of CV-A2-infected children. In another enterovirus epidemic in Hangzhou in 2015, China, CV-A2 was found to be the major pathogen of herpangina^21^. Among 2,310 cases analyzed, 1651 were enterovirus positive (71.5%), and of 35 serotyped samples, 22 were CV-A2. In this large scale of outbreak, involving 10,210 children diagnosed as HA, only 35 cases were used for sequencing and serotyping unfortunately. In our study, serotyping was performed on all 4415 cases of HFMD, and it was found that CV-A2, as well as CV-A5, detected in a high proportion and closely linked with HFMD.

This study began in October accompanied with a large scale of vaccination of inactivated EV-A71 vaccine from September to October 2016 in Xiangyang City. A sharp decrease of EV-A71 HFMD cases with increase of CV-A2 and CV-A5 cases was observed in beginning of 2017, 2- to 3-months post-vaccination. This may be explained why the proportions of these CV-A2 and CV-A5 were higher than EV-A71 in 2017. Importantly, this was a whole spectrum etiological study following a large sale vaccination of inactivated EV-A71 vaccine. It seems that EV-A71 vaccine reduced the HFMD cases and perhaps reduced the severe cases caused by EV-A71 infection as shown in Table 2. However, the total cases of HFMD largely remained the same at the winter peaks. In this study, most of severe cases were caused by non-EV-A71 HEVs. The inactivated EV-A71 vaccine reduced mild, severe cases caused with EV-A71, but other serotypes were still circulating, emerging and causing severe cases of HFMD. Unchanged total cases and severe cases caused by non-EV-A71 HEVs suggest the urgent need for tetravalent or even hexavalent vaccines including antigen components of EV-A71, CV-A16, CV-A6, CV-A10, CV-A5 and CV-A2.

The high mutation rate of enteroviruses, like other RNA viruses, have been observed due to the low fidelity of their RNA-dependent RNA polymerase and the absence of mismatch repair mechanism^26^. Ogi M and colleagues found that CV-A6 isolates before 2009 and after 2011 were divided into two clusters based on sequence analysis, which were mostly related to HA and HFMD, respectively. The accumulation of point mutations of CV-A6 might be associated with the clinical course^7^. Also for CV-A6, mutations may cause atypical HFMD in adult patients^13^. Feng and colleagues reported that a recombinant CV-A6 led to a more generalized rash than the non-recombinant CV-A6 and may be also related to the emerging and spreading of this recombinant^27^. Co-circulation of six main serotypes might increase frequency of co-infection and therefore intertype recombination. Further study on mutations and recombination of CV-A2 and CV-A5 viruses based on the complete sequences of these two serotypes is underway to explain the emerging and transmission of these two serotypes. Continued surveillance of CV-A2, CV-A5 and other serotypes which related to HFMD and other diseases, is important to monitor the potential change of etiological spectrum and disease patterns in China and worldwide.

All echoviruses were isolated in RD cells but not in Vero cells, consistent with previous report^28–34^. It is known that different enterovirus serotypes may be sensitive to different cell lines and presumptive identification of enteroviruses has been performed previously on several cell lines^35–37^. In this study, it was found that presumptive identification of some serotypes with cell culture such as CV-A2 and CV-A5, CV-A6 and CV-A10 was difficult as the isolation rates in RD, Vero, MRC-5, 2BS were very low (data not shown). Also, it was observed that false CPE positive was high if only one passaging of cell cultures was performed (15–20% of CPE positive, data not shown). However, cell cultures helped to identified co-infections of patients with two serotypes. Co-infection with CV-A16 and EV-A71 can cause serious complications in the central nervous system (CNS) and increase the chance of intertype recombination, which may be responsible for the large HFMD outbreak in Mainland China in 2008^38,39^. A multivalent HFMD vaccine is required for broad protection against HFMD as there are no cross-neutralizing activity between antisera against different serotypes of HFMD-associated enteroviruses. The inclusion of CV-A16, CV-A6, CV-A10 and EV-A71 in a multivalent HFMD vaccine is rational and feasible^40^.

In this study, a combination of three approaches was used for serotyping of all HEV positive specimens of HFMD patients collected in 15 months. The Real Time RT-PCR is rapid and conventional PCR/sequencing helps to analyze genotype and subgenotype. Interestingly, it was found that conventional RT-PCR was more sensitive than Real Time RT-PCR for detection of some of serotypes of HEVs in this study, consistent with the results previously reported^41^. Cell culture is a timing consuming method, but it is very important to obtain strains for further study on molecular biology of these viruses and for development of vaccine and therapeutic agents. In fact, it seems that some enterovirus serotypes are more easily identified by using cell cultures than using the other two methods, as demonstrated in this study. By using Real Time RT-PCR and conventional RT-PCR, 12 serotypes were detected, while cell cultures helped to identify the other 6 serotypes. Li found no significant association between EV-A71 vaccination and CV-A6 or CV-A16-related hand, foot, and mouth disease^42^.

In conclusion, the molecular epidemic study indicated that etiological spectrum of HFMD have been changing. The EV-A71 monovalent vaccine may decrease severe and fatal cases of HFMD. However, to prevent of the disease a multivalent vaccine is urgently needed. Currently, a tetravalent vaccine including EV-A71, CV-A6, CV-A10 and CV-A16 could cover approximately 90% of pathogens associated with HFMD in China.

## Materials and methods

### Ethical approval and informed consent

This study protocol was approved by the medical ethics committee of the Wuhan Institute of Biological Products and Huazhong University of Science and Technology (201609). Patients or their guardians were informed of the information of this study and informed consent was obtained from guardians of all subjects. All experiments were performed in accordance with relevant guidelines and regulations.

### Case definitions

HFMD cases were diagnosed according to the criteria issued by the National Health and Family Planning Commission, People’s Republic of China. Suspect HFMD cases were defined as patients showed symptoms of maculopapular or vesicular rash on the hand, foot, mouth, and buttocks with or without fever. Severe HFMD cases were defined as those cases with neurological complications such as aseptic meningitis, encephalitis, encephalomyelitis, acute flaccid paralysis, or dysfunction of the autonomic nervous system, and cardiopulmonary complications (pulmonary edema, pulmonary hemorrhage), or cardiorespiratory failure, or both. Otherwise, patients were categorized as mild cases.

Laboratory confirmation was performed for serotyping of all specimens. Firstly, cases diagnosed as HFMD were confirmed in laboratory with detection of enterovirus RNAs using EV-71 specific and pan-enterovirus Real Time RT-PCR Kits (Shanghai ZJ Bio-Tech Co, Ltd, Shanghai, China). Secondly, EV-71 negative and enterovirus positive (EV-71−/HEV+) specimens and pan-enterovirus primers were used for amplification and sequencing of the partial 5′-UTR and the VP1 regions, and followed by serotyping/genotyping.

### Specimen collection

Rectal swabs were collected from children with the symptoms of HFMD. The specimens were collected from inpatients and outpatients in three hospitals in Xiangyang, Hubei, China, in viral transport medium which was purchased from Youkang, Beijing, China and containing Hanks balanced salt solution, HBSS. The specimens were stored at − 80 °C for subsequent viral RNA extraction and isolation of viruses in cell cultures.

### Nucleotide acid detection by real time RT-PCR

Viral RNAs were extracted using nucleic acid automatic extraction instrument (Tianlong Science & Technology, Jiangsu, China). The detection of HEV was performed in Applied Biosystems-7500 system by one-step Real-Time RT–PCR assay with specific Enterovirus 71 and Pan-Enterovirus Real Time RT-PCR Kits (Shuoshi Biotech, Jiangsu, China). The Real Time RT-PCR was conducted under conditions of 30 min at 50 °C by 1 cycle, 5 min at 95 °C by 1 cycle, and then followed by 45 cycles with 10 secs at 95 °C and 40 secs at 55 °C. Samples with CT value less than 43 were considered positive.

### Amplification of the partial 5′-UTR and VP1 regions and sequencing

The cDNA was synthesized by using PrimeScript 1st Strand cDNA Synthesis Kit (TakaRa, Dalian, China) in a total volume of 20 μL with 5 μL of RNA. The amplification was performed by nested-PCR using the 5′-UTR or VP1 primers of the enteroviruses, as described previously^43^. For the first round PCR, 2 μL of cDNA was used as a template with 1 μL each of outer primers (10 uM/μL), 10 μL Premix Taq (TakaRa, Dalian, China) in a volume of 20 μL following cycling conditions of 94 °C pre-denaturation for 2 min, 40 cycles of 94 °C for 15 s, 55 °C for 30 s, 68 °C for 40 s, then 68 °C extension for 5 min. A volume of 1 μL of the product was used for the second round of PCR with 1 μL each of inner primers (10 μM/μL) in a volume of 20 μL under the same PCR conditions described above. Furthermore, representative strains of each serotype were amplified by 292 and 222 primers, as described previously^44^, to build a phylogenetic tree based on the VP1 gene. The final PCR products were subjected to 1.0% agarose gel electrophoresis for analysis followed by sequencing.

### Phylogenetic analysis

The genetic identity of each PCR product was first determined by comparison with the reference strains in GenBank (National Center for Biotechnology Information, NCBI). Phylogenetic trees based on the partial VP1 genes were built using the neighbor-joining method in the Molecular Evolutionary Genetics Analysis program, version 6.0 (MEGA 6.0). Reference strains of CV-A6, CV-A16, CV-A10, CV-A5, CV-A2 and EV-A71 were retreated from GenBank and were aligned with the sequences of individual serotypes.

### Virus isolation in cell cultures

African green monkey kidney (Vero) and human rhabdomyosarcoma (RD) cells were grown in Dulbecco’s modified Eagle’s medium (DMEM) supplemented with 10% of fetal calf serum (Gibco, USA), 100 U/mL penicillin and 0.1 mg/mL streptomycin. RD and Vero cells were used to isolate viruses in rectal swab specimens by blindly passaging 3 times. The CPE positive cultures were further passaged once and enteroviruses were identified by the conventional RT-PCR amplification and sequencing of the partial 5′-UTR and VP1 genes using pan-HEV primers as described above. The complete nucleotide sequences of representative strains of the six major serotypes were also performed.

## Supplementary information

Supplementary Information.

## Data Availability

Please contact corresponding author for data requests.
