# Supplementary material for: Epidemical and etiological study on hand, foot and mouth disease following EV-A71 vaccination in Xiangyang, China
Source: Sci Rep. 2020 Dec 1;10:20909. doi: 10.1038/s41598-020-77768-7 (PMC7708472; doi:10.1038/s41598-020-77768-7)
Supplement: Supplementary file 1 — Supplementary Information. [file 41598_2020_77768_MOESM1_ESM.docx]

**Epidemical and etiological study on hand, foot and mouth disease following EV-A71 vaccination in Xiangyang, China**

**Xiao-Dan Meng ^1+^, Yeqing Tong^2+^, Zhen-Ni Wei ^1+^, Lei Wang^2^, Jian-Yi Mai^1^, Yang Wu^2^, Zhi-Yu Luo^1^, Shaoping Li^3^, Meng Li^1^, Siquan Wang^2^, Sheng Wei^4^, Wensheng Gong^3^ , Wangsheng Zhang^3^, Xingzhou Hu^3^, Jiao Huang^4^, Jing Shi^3^, Gang Yang^3^, Sheng-Li Meng^1^，****Ze-Jun Wang^1^*, Xuhua Guan^2^*, Shuo Shen^1^***

| Year/Month | EV-A71 | Other HEVs | HEV negative | Severe case |
| --- | --- | --- | --- | --- |
| 2016.10 | 14 | 103 | 55 | 0 |
| 2016.11 | 21 | 198 | 112 | 1 |
| 2016.12 | 13 | 254 | 84 | 2 |
| 2017.01 | 6 | 189 | 50 | 2 |
| 2017.02 | 0 | 95 | 38 | 1 |
| 2017.03 | 1 | 305 | 46 | 2 |
| 2017.04 | 1 | 525 | 48 | 7 |
| 2017.05 | 4 | 238 | 38 | 1 |
| 2017.06 | 8 | 247 | 32 | 4 |
| 2017.07 | 6 | 200 | 35 | 2 |
| 2017.08 | 2 | 131 | 13 | 0 |
| 2017.09 | 0 | 178 | 12 | 0 |
| 2017.10 | 2 | 311 | 21 | 0 |
| 2017.11 | 0 | 427 | 19 | 0 |
| 2017.12 | 0 | 302 | 31 | 0 |
| No. of samples | 78 | 3,703 | 634 | 22 |

**Supplementary Table S1.** Monthly distribution of total and severe HFMD cases Total number of HFMD cases caused by EV-A71, other HEVs and non-HEV (HEV negative) were 4,415 and were confirmed using Real Time PCR Kits, specific EV-A71 and pan-HEVs.

| Year/Month | CV-A6 | CV-A16 | CV-A10 | CV-A5 | CV-A2 | EV-A71 |
| --- | --- | --- | --- | --- | --- | --- |
| 2016.10 | 53 | 23 | 3 | 0 | 0 | 15 |
| 2016.11 | 113 | 39 | 15 | 0 | 0 | 25 |
| 2016.12 | 176 | 10 | 43 | 0 | 2 | 23 |
| 2017.01 | 120 | 7 | 28 | 1 | 0 | 8 |
| 2017.02 | 87 | 6 | 10 | 0 | 0 | 0 |
| 2017.03 | 190 | 8 | 35 | 5 | 3 | 2 |
| 2017.04 | 205 | 30 | 82 | 28 | 14 | 4 |
| 2017.05 | 125 | 25 | 49 | 32 | 25 | 5 |
| 2017.06 | 97 | 48 | 37 | 23 | 10 | 7 |
| 2017.07 | 53 | 22 | 26 | 23 | 18 | 4 |
| 2017.08 | 42 | 18 | 9 | 8 | 6 | 2 |
| 2017.09 | 71 | 19 | 9 | 7 | 10 | 0 |
| 2017.10 | 210 | 48 | 12 | 13 | 20 | 2 |
| 2017.11 | 247 | 94 | 10 | 6 | 13 | 0 |
| 2017.12 | 117 | 93 | 2 | 0 | 0 | 0 |
| No. of samples | 1,906 | 490 | 370 | 146 | 121 | 97 |

**Supplementary Table S2.** Monthly distribution of HFMD cases caused by the six major serotypes. A total number of HFMD cases caused by the six major serotypes is 3,130, confirmed with combination of Real Time RT-PCR, conventional RT-PCR and cell cultures.
